# The Role of Micronutrients in Graft-VS.-Host Disease: Immunomodulatory Effects of Vitamins A and D

**DOI:** 10.3389/fimmu.2018.02853

**Published:** 2018-12-06

**Authors:** Xiao Chen, Christopher G. Mayne

**Affiliations:** ^1^Division of Hematology & Oncology, Department of Medicine, Medical College of Wisconsin, Milwaukee, WI, United States; ^2^Biology Department, Viterbo University, La Crosse, WI, United States

**Keywords:** vitamin A, vitamin D, retinoic acid, vitamin D receptor, graft-vs.-host disease, allogeneic hematopoietic stem cell transplantation

## Abstract

Graft-vs.-host disease (GVHD) remains a major obstacle to the success of allogeneic hematopoietic stem cell transplantation (HSCT). GVHD occurs because donor T cells in the allograft recognize the genetically disparate host as foreign and attack the transplant recipient's tissues. While genetic incompatibility between donor and recipient is the primary determinant for the extent of alloimmune response, GVHD incidence and severity are also influenced by non-genetic factors. Recent advances in immunology establish that environmental factors, including dietary micronutrients, contribute significantly to modulating various immune responses and may influence the susceptibility to autoimmune and inflammatory diseases of experimental animals and humans. Emerging clinical and preclinical evidence indicates that certain micronutrients may participate in regulating GVHD risk after allogeneic HSCT. In this review, we summarize recent advances in our understanding with respect to the potential role of micronutrients in the pathogenesis of acute and chronic GVHD, focusing on vitamins A and D.

## Micronutrients and Immunity

Micronutrients are compounds that are only needed in small amounts, yet are essential for the proper growth and development of the human body. These vitamins and minerals are indispensible for the production and function of various enzymes and hormones that are critical for maintaining optimal physical and mental function. An aberrant micronutrient status contributes to the increased susceptibility to various infectious, inflammatory, and metabolic conditions such as colitis, diabetes, cancer, obesity, and cardiovascular disease.

The importance of the micronutrients vitamins A and D in health has been recognized since the early twentieth century. More recent advances have led to the discovery of the critical role of these molecules in the immune system (1). Current highlights within this field include the finding that maternal vitamin A levels significantly influence the proper development of secondary lymphoid organs in offspring and determine the fitness of their immune system in later life (2). Lack of vitamin A-mediated signaling in utero substantially reduced the anti-pathogen immune response of newborn mice (2). Similarly, vitamin D also plays a role at the maternal-fetal interface, preventing inflammatory responses such as pre-eclampsia (3). These immunomodulatory effects may be long lasting as maternal vitamin D deficiency has been shown to contribute to a greater likelihood of atopic responses in the neonatal lung (4, 5). These findings reveal how nutritional status during fetal life can profoundly affect immune responses in adulthood, highlighting the importance of vitamins A and D in the development and maintenance of a competent, yet tightly regulated immune system.

## Graft-VS.-Host Disease (GVHD) and Nutritional Factors

**Figure 1:**
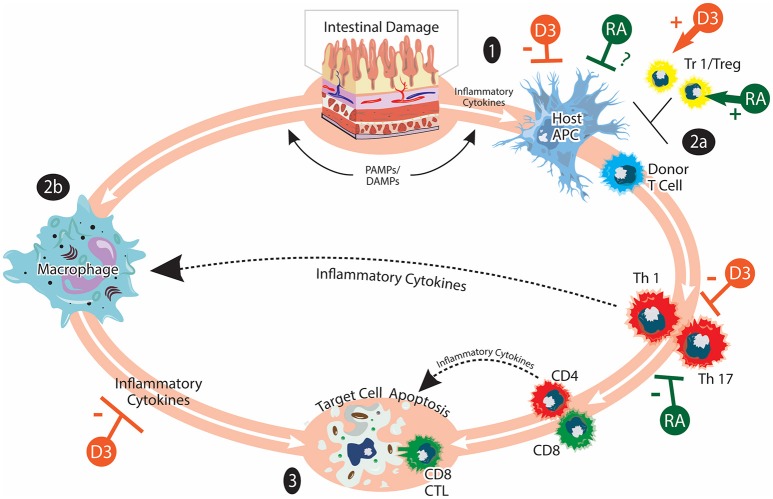
Acute GVHD and vitamins A and D. aGVHD pathogenesis involves: (1) Activation of host APCs due to release of inflammatory cytokines and PAMPs/DAMPs from tissue damaged by HSCT conditioning. Intestinal damage by conditioning serves to amplify inflammatory responses. (2) Activation of donor T cells when they encounter host APC. Donor T cells undergo differentiation, expansion, and acquisition of tissue homing specificity during this stage (2a). Inflammatory cytokines produced by donor T cells and bacterial LPS can further activate innate immune cells such as macrophages (2b). (3) Inflammatory mediators from donor T cells and innate immune cells lead to target cell apoptosis. Cytotoxic CD8 T cells can mediate direct cell killing. Tr1/Tregs play immunomodulatory roles in aGVHD pathogenesis. The effects of vitamin A/RA (shown in green) on aGVHD are complex and not completely understood. Vitamin A/RA promotes donor T-cell intestinal homing. Inhibiting donor T-cell RAR signaling suppresses the induction of gut-homing molecules and favors Treg cell differentiation. It has also been reported that RA inhibits donor T cell expansion and cytokine production. The potential effects of vitamin A/RA on host APCs are currently under investigation. The effects of vitamin D (shown in orange) on aGVHD may include suppressing the activation of host APCs, inhibiting the activation and cytokine production of donor T cells as well as promoting the induction of Tr1/Treg. The figure is adapted from Ferrara et al. (9).

**Figure 2:**
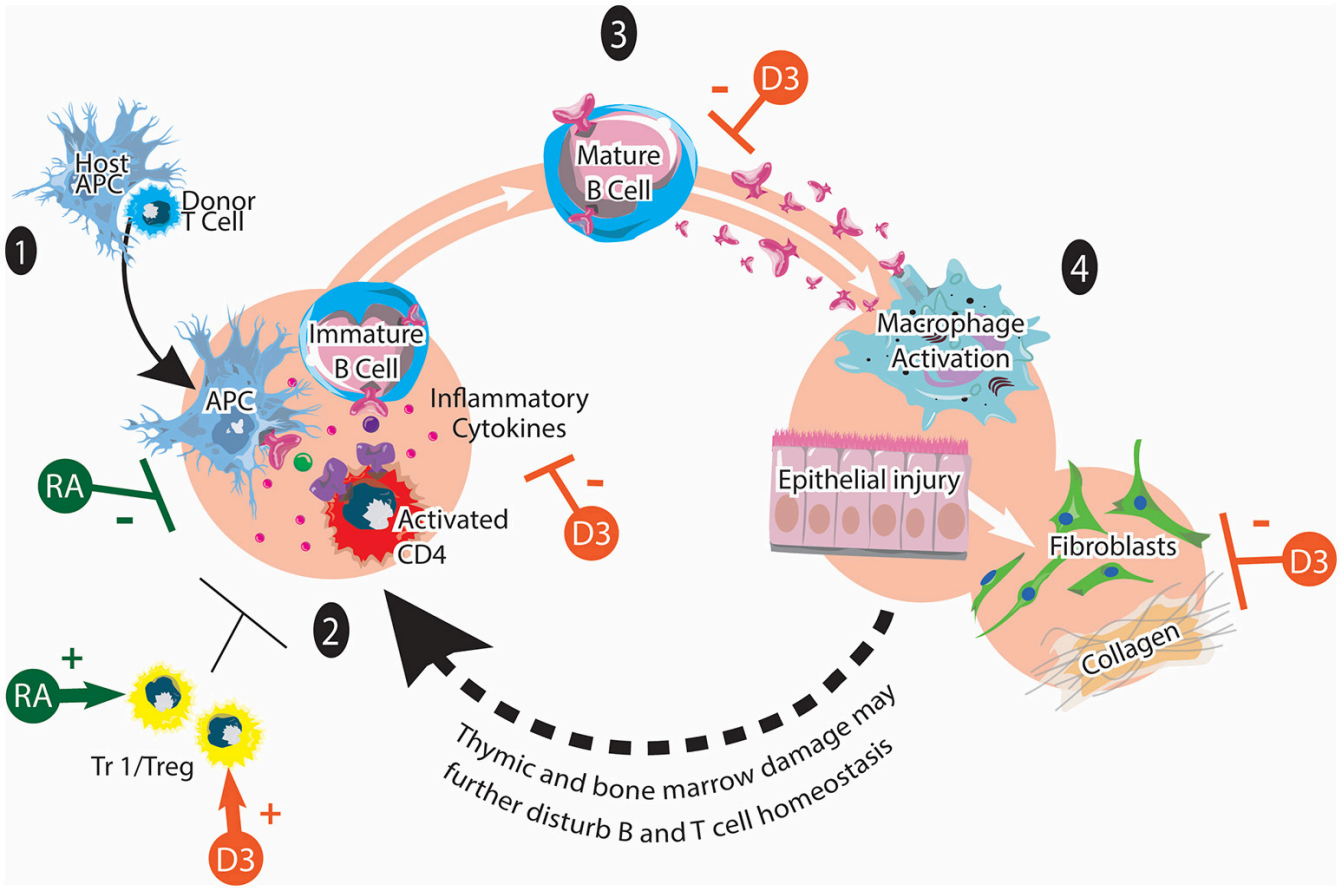
Chronic GVHD and vitamins A and D. cGVHD pathogenesis involves: (1) Early inflammation and tissue injury. An existing inflammation and danger signals activate innate immune cells and recruits donor T cells to the tissue. (2) Dysregulated immunity with loss of tolerance. Activated CD4 T cells stimulate the maturation of auto-reactive B cells. (3) Mature B cells produce various autoantibodies against host antigens. (4) Aberrant tissue repair and fibrosis via macrophages and fibroblasts. Tr1/Treg play immunomodulatory roles in chronic GVHD pathogenesis. It has been reported that synthetic retinoid (shown in green) reduces cGVHD by inhibiting Th1 and Th17 cells. It may also facilitate the generation of Tr1/Tregs. The potential effects of vitamin D on cGVHD (shown in orange) may include positive effects on Tr1/Treg function and polarization as well as inhibitory effects on proinflammatory T cell polarization, inflammatory cytokines, autoantibody secretion and collagen production. This figure is adapted from Cooke et al. (12).

GVHD remains a major obstacle limiting the broader application of allogeneic hematopoietic stem cell transplantation (HSCT), an effective treatment for a number of malignant and non-malignant hematological disorders (6–8). GVHD is the consequence of a normal, yet exaggerated, immune reaction elicited by donor T cells when they encounter alloantigens expressed by the transplant recipient. Acute GVHD (aGVHD) pathophysiology is characterized by strong inflammatory components while chronic GVHD (cGVHD) displays more autoimmune manifestations (9–13). The pathogenesis of GVHD is a complex process involving a variety of host and donor immune cells (Figures 1, 2). The major determinant for the development and the severity of GVHD is the genetic disparity between the donor and recipient. However, some non-genetic factors such as the level of exposure to damage associated molecular patterns (DAMPs) and pathogen-associated molecular patterns (PAMPs) are also important components of GVHD pathophysiology, due to their ability to amplify inflammatory responses (14). In addition, other host factors may also influence the function of various immune cells and modulate the alloimmune response.

Nutritional status is a significant variable among patients undergoing allogeneic HSCT. In fact, nutritional support appears to affect the development of GVHD, with adequate enteral nutrition being associated with reduced GVHD risk as compared to parenteral nutrition (15–18). These studies indicate that the interaction between certain oral nutrition and the gastrointestinal tract can modulate GVHD risk. Thus, patient nutritional status may be an independent and modifiable factor influencing GVHD severity (19). It is conceivable that an improved nutritional status may provide patients with an increased ability to tolerate treatment-associated toxicity and recover from GVHD-associated tissue damage. More importantly, certain micronutrients may also be actively involved in regulating the initiation, development, and resolution of inflammatory responses after HSCT. In this review, we briefly summarize the potential roles of vitamins A and D in GVHD pathogenesis.

## Effects of Vitamin A on GVHD

Vitamin A is a multifunctional vitamin involved in a wide range of biological processes. Most biological effects of vitamin A are exerted by its major metabolite, retinoic acid (RA) (20). The conversion from vitamin A to RA requires two hydrolysis steps catalyzed first by alcohol dehydrogenases (ADHs), followed by aldehyde dehydrogenases (RALDHs). RALDHs are the rate-limiting enzymes for RA synthesis and are expressed in limited tissues (21). Heterodimers of retinoic acid receptors (RARs) and retinoid X receptors (RXRs) mediate RA signaling. These heterodimers bind to retinoic acid responsive elements (RARE) of target genes and regulate gene transcription. One of the most important physiological functions of vitamin A and RA is to regulate immune responses, and dysregulated retinoid signaling can lead to a weakened immunity against pathogens and/or the loss of immune homeostasis (20, 22).

RA has pleiotropic effects on cells of the innate and adaptive immune system (23–25). It can target T cells, B cells, antigen presenting cells (APCs), and innate lymphoid cells (ILCs) to regulate immune responses. RA induces the expression of gut-homing molecules CCR9 and α4β7 on various immune cells, augmenting cell migration to the intestines (26–28). At steady state, RA promotes the induction of tolerogenic dendritic cells (DCs). However, in the presence of inflammatory cytokines such as IL-15, RA promotes the induction of inflammatory DCs and intensifies pathogenic mucosal immune responses (29). RA has also been shown to influence the development of DC subsets in the spleen and intestines (30–33). RA also plays a central role in modulating intestinal CD4^+^ T cell responses and enhances the stability of natural regulatory T cells (Tregs) (34). Together with TGF-β, RA promotes the conversion of naïve T cells into induced-Tregs at the expense of Th17 cells (35, 36). Vitamin A deficiency is associated with impaired oral tolerance, suggesting an important role of RA in maintaining intestinal homeostasis (37, 38). On the other hand, the RA-RAR-α axis is important for CD4^+^ T cell activation and effector function under inflammatory conditions (39, 40). Finally, RA promotes the induction of ILC3 but suppresses the generation and cytokine production of ILC2 (41). These observations demonstrate the complex and sometimes paradoxical functions of RA within the immune system, as it can possess either pro-inflammatory or anti-inflammatory properties depending on the context.

In the context of GVHD, Koenecke and colleagues used a dietary approach to first examine how recipient vitamin A levels affect donor T cell trafficking after experimental HSCT (42). Vitamin A-deficient (VAD) recipient mice had a reduced percentage and absolute number of donor T cells in the intestine, which was attributable to diminished expression of gut-homing molecules α4β7 and CCR9. VAD recipients survived longer than control vitamin A-normal (VAN) mice due to gastrointestinal protection, though they developed more severe hepatic GVHD. These results indicated that vitamin A affects GVHD target organ tropism of donor T cells, with a particularly important role in controlling the migration of donor T cells to the intestine, a critical GVHD target organ (43). We and others then used a genetic approach to examine the role of RA signaling in GVHD pathogenesis. These studies consistently showed that genetic ablation of RAR-α on donor T cells significantly decreased the ability of these cells to cause lethal GVHD (44, 45). This was largely due to reduced expression of gut-homing molecules CCR9 and α4β7 on donor T cells with diminished intestinal migration. In contrast, administrating RA exogenously to recipient mice increased expression of gut-homing molecules on donor T cells and increased their gut-tropism, leading to a significantly increased overall mortality (44–46). In addition, inhibiting RAR-α reduced donor T cell differentiation toward a Th1 phenotype and favored the induction of Tregs (45), which also contribute to the decreased ability of these cells to cause GVHD. Importantly, genetic inhibition of RAR-α signaling on donor T cells does not compromise their ability to mediate the graft-vs.-leukemia effect.

In an effort to improve the translational potential of this research, we treated donor mice with BMS493, a pan-RAR antagonist. Recipients of BMS493-treated donor T cells showed improved overall survival after HSCT compared to recipients of vehicle-treated donor T cells, indicating that pharmacological inhibition of the retinoic acid pathway on donor T cells can reduce their alloreactivity and ability to cause GVHD (47). Interestingly, chronic vitamin A deficiency changed the composition of the donor T cell compartment with a reduction in the percentage of CD4^+^ T cells, resulting in reduced ability of transferred T cells from VAD mice to cause lethal GVHD (47). Thus, both host and donor vitamin A levels appear to affect the development of experimental GVHD (42, 47). While most preclinical studies suggest a detrimental effect of RA on GVHD, it has also been reported that RA treatment reduces aGVHD (48) and a synthetic retinoid ameliorates cGVHD (49). Differences in mouse GVHD models used, RA levels *in situ*, and local cytokine milieu could all potentially contribute to these differing observations.

Apart from above preclinical studies, emerging clinical data also demonstrate the involvement of vitamin A/RA in GVHD pathogenesis. A recent study found that lower levels of vitamin A are associated with increased intestinal GVHD in children receiving allogeneic HSCT (50). The incidence of grades 2–4 GVHD was also significantly higher in patients with lower vitamin A levels. These observations appear in contrast to a murine study in which vitamin A deficiency is associated with a reduced intestinal GVHD and improved overall survival (42). This discrepancy could be due to inherent differences between mouse model and human disease or increased severity of experimentally induced vitamin A deficiency compared to clinical deficiency/insufficiency. In addition, the study by Lounder et al. actually used serum vitamin A levels above or below a median value, instead of vitamin A deficiency or sufficiency, to separate patient groups. Finally, there was also evidence that low serum vitamin A levels are associated with more severe ocular GVHD in allogeneic HSCT patients (51). Thus, both preclinical and clinical data indicate a significant involvement of vitamin A and RA pathway in GVHD pathogenesis.

## Effects of Vitamin D on GVHD

Vitamins D and A are similar in that they are the only two vitamins whose active metabolites have hormone-like properties. Indeed, the active metabolite of vitamin D, calcitriol, is a well-established secosteroid hormone with multiple roles throughout the human body (52). Though vitamin D may be acquired nutritionally, a large proportion of vitamin D is synthesized in the human body. This synthesis is initiated in the skin as UV-B rays cause the photolysis of 7-dehydrocholesterol, forming vitamin D_3._ In the liver, vitamin D_3_ is hydroxylated to 25-hydroxyvitamin D_3_ (25(OH)D_3_) by enzymes such as CYP2R1 and CYP27A1 (53, 54). 25-hydroxyvitamin D_3_ is the principal circulating metabolite of vitamin D and 25(OH)D_3_ concentration is typically used as an indicator of vitamin D status. This inactive 25(OH)D_3_ is hydroxylated once more in the kidney via the enzyme CYP27B1 to become the biologically active hormone 1,25-dihydroxyvitamin D_3_ (1,25(OH)_2_D_3_), also known as calcitriol.

Vitamin D utilizes similar signaling mechanisms to vitamin A. Calcitriol binds to the vitamin D receptor (VDR), which heterodimerizes with RXR (55, 56). VDR-RXR heterodimers that are bound to calcitriol act as transcriptional regulators by binding vitamin D response elements (VDREs) of target genes (57). The classical physiological roles of vitamin D (via calcitriol) are in calcium and phosphate homeostasis and bone metabolism, with other roles being referred to as “non-classical” functions. The discovery of vitamin D binding within immune cells in the early 1980s and eventual description of VDR expression in immune cells were key steps in the study of the non-classical effects of vitamin D on the immune system (58–60).

It has been shown in numerous studies that calcitriol inhibits maturation and inflammatory cytokine production of DCs (61–64). These changes in DC differentiation and function also result in a skew toward a more tolerogenic DC profile with the ability to drive Treg, T-regulatory cell type 1 (Tr1), and Th2 cell development (65). Importantly, calcitriol also appears to exhibit direct effects on CD4 T cell populations to modify immune function. *In vitro* treatment of T cells with calcitriol inhibits proliferation under several activating conditions (66, 67). Calcitriol has been shown to be effective as a treatment in numerous mouse models of diseases that are driven by Th1 and Th17 cells, suggesting a global immunomodulatory effect on these cell types (68–71). Calcitriol also appears to inhibit proliferation and pathogenicity of CD8 T cells since VDR-deficient CD8 T cells are hyperproliferative and proinflammatory (72, 73). Calcitriol inhibits production of IFN-γ and stimulates IL-4 secretion in invariant natural killer (iNKT) cells (70). Finally, calcitriol leads to decreased B cell proliferation and differentiation to plasma cells. However, it is unclear if these effects are due to direct effects on the B cell, or due to reduced interactions with CD4 T cells (74).

There is a significant history of studies on the effects of vitamin D and its analogs on allograft survival in several tissues (75, 76). However, preclinical studies in animal models of allogeneic HSCT are rather limited. To our knowledge, only one study of VDR agonism has been reported in animal models of GVHD. In this study, a vitamin D analog reduced aGVHD severity and immune cell infiltration in liver, skin, and spleen of rats (77). *In vitro* studies utilizing human monocyte-derived DCs recapitulated previously results showing that vitamin D led to the development of more immature tolerogenic DCs. Vitamin D-treated DCs activated allogeneic CD4 and CD8 T cells with a greater IL-10 to IFN-γ ratio, and these T cells were less proliferative in mixed lymphocyte reactions (MLRs) (78). In another study, alloreactive T cells were shown to express greater levels of VDR (79). Addition of calcitriol to the MLR led to a decrease in the percentage of proliferating T cells. This appeared to be due to direct action of calcitriol on the alloreactive T cells since the allogeneic DC were matured in the absence of calcitriol and irradiated prior to be used in the MLR (79).

The first reports for a potential role of vitamin D in human GVHD came through candidate gene studies analyzing known *VDR* polymorphisms. Interestingly, the results of these studies are quite variable. Some studies suggest roles for various polymorphisms in GVHD when present in the recipient only (80–82), some studies suggest a role for VDR genotype in both the donor and recipient (83), while another more recent study found no significant association between GVHD and VDR polymorphisms in the donor nor recipient (84). Taken together, these results suggest that in some instances the *a* allele of *VDR* may play a role in aGVHD risk when present in recipients of HSCT. However, not all studies have found such an association and the results may vary between different populations (84). One complicating factor of these studies was that the vitamin D status of the individuals studied was often unknown. Thus, any differences in VDR activity associated with disease could be obfuscated depending on whether an individual's vitamin D stores were sufficient to provide for VDR function.

Though genetic studies of *VDR* suggest a potential role for vitamin D signaling in GVHD, patient serum levels of vitamin D may provide a more direct method of investigation. Indeed, it appears that individuals undergoing HSCT are at particular risk for vitamin D deficiency/insufficiency (85–89). Several retrospective studies have thus investigated whether vitamin D status prior to HSCT corresponds with subsequent development of GVHD (90–92). These studies seem to suggest a relatively consistent association of cGVHD with lower vitamin D status, whereas the results for aGVHD are more variable. In a more recent study, however, levels of vitamin D pre-HSCT did not correlate to development of aGVHD nor cGVHD in a group of pediatric patients (93). Interestingly, the one-year survival rate did differ significantly, with 35% mortality in the deficient group vs. 0% in the insufficient and 7% in the sufficient groups, suggesting a beneficial effect of higher vitamin D levels on overall survival after allogeneic HSCT (93).

Given the potential association of vitamin D status and GVHD, the effect of supplementation was further investigated. Even though HSCT patients may be particularly at risk for vitamin D insufficiency/deficiency, supplementation can increase their vitamin D status (86, 90). Rosenblatt et al. reported observation of two patients with steroid refractory cGVHD who were treated with supplemental vitamin D for bone mineral abnormalities. Impressively, both had their symptoms wane to the point that they were removed from immunosuppression after vitamin D treatment (78). A marked improvement in cGVHD was also observed in a subsequent analysis of 12 adult HSCT patients who were given 1,000 IU/day vitamin D to treat osteopenia or osteoporosis (94).

To date, we are aware of only one published prospective study of vitamin D supplementation in HSCT patients (95). This investigation demonstrates that vitamin D may play a role in the prevention of cGVHD, as suggested previously (78, 90–92, 94). Intriguingly, there was no difference in aGVHD among the patient groups in this study. It is worth noting that in this study vitamin D supplementation began only 3 days prior to transplantation. Indeed, the authors show that significantly higher levels of 25(OH)D_3_ were not observed in the serum until day 7 in high-dose and day 21 in low-dose patients (95). This signifies that aGVHD may have been initiated in the absence of sufficiently elevated levels of vitamin D. Further investigation into the effect of earlier supplementation to raise serum vitamin D levels prior to HSCT to prevent aGVHD is of interest. Overall, the data surrounding vitamin D and the immune system as well as the initial studies on vitamin D and GVHD suggest that it is highly likely that vitamin D could exhibit positive effects in the prevention and/or treatment of GVHD (96).

### Conclusions and Perspectives

In conclusion, we believe that small molecules like vitamins A and D could have the potential to influence the development of GVHD after allogeneic HSCT. These micronutrients may modulate crosstalk between the various immune cells involved in the pathogenesis of GVHD, thus influencing disease initiation, progression, and resolution (Figures 1, 2). Their levels may also have prognostic value, serving as independent risk factors for predicting the severity of organ-specific or systemic GVHD. Most importantly, nutritional intervention before and after allogeneic HSCT may be used as an adjuvant therapy to reduce GVHD risk and improve the outcome of allogeneic HSCT (95, 97, 98). We propose that GVHD research using animal models should consider dietary composition. More preclinical studies in this understudied research area will provide new insights into how nutritional factors contribute to GVHD pathogenesis. Finally, more prospective randomized controlled clinical trials are needed to fully reveal the potential of using micronutrients such as vitamins A and D as simple and inexpensive approaches with minimal side effects to mitigate clinical GVHD.

## Author Contributions

All authors listed have made a substantial, direct and intellectual contribution to the work, and approved it for publication.

### Conflict of Interest Statement

The authors declare that the research was conducted in the absence of any commercial or financial relationships that could be construed as a potential conflict of interest.
